# A Case of Refractory Hypothyroidism due to Poor Compliance Treated with the Weekly Intravenous and Oral Levothyroxine Administration

**DOI:** 10.1155/2019/5986014

**Published:** 2019-02-05

**Authors:** Yujiro Nakano, Koshi Hashimoto, Noriaki Ohkiba, Hideyuki Okuma, Isao Minami, Hiromitsu Takahashi, Yuji Tanaka, Takanobu Yoshimoto, Tetsuya Yamada

**Affiliations:** ^1^Department of Molecular Endocrinology and Metabolism, Graduate School of Medical and Dental Science, Tokyo Medical and Dental University, 113-8510, Japan; ^2^Department of Preemptive and Metabolism, Graduate School of Medical and Dental Science, Tokyo Medical and Dental University, 113-8510, Japan; ^3^Department of Endocrinology and Metabolism, Kashiwa Municipal Hospital, 277-0825, Japan; ^4^Department of Pharmacy, Medical Hospital of Tokyo Medical and Dental University, 113-8510, Japan; ^5^Department of General Medicine, National Defense Medical College Hospital, 359-0042, Japan

## Abstract

Refractory hypothyroidism is caused by decreased gut absorption, increased metabolism, and poor compliance. Previous studies suggested that the weekly oral, suppository, or intramuscular administration of levothyroxine (LT4) is an effective treatment for refractory hypothyroidism. However, limited information is currently available on treatment involving the weekly intravenous administration of LT4. We managed a case of refractory hypothyroidism due to poor compliance, for which, by weekly intravenous LT4 administration, LT4 was intravenously administered weekly at a dose of 300 *μ*g without any adverse effects such as acute ischemic heart diseases or liver dysfunction and effectively maintained the euthyroid status for 14 months. The weekly oral administration of LT4 (700 *μ*g) was also safely performed and was as effective as its intravenous administration. We herein present precise clinical course of the present case including pharmacokinetic data during the weekly intravenous and oral administration of LT4 and discuss the safety and efficacy of the treatments.

## 1. Introduction

Hypothyroidism is one of the most common thyroid disorders, which more frequently affects women, and its incidence increases with age [1]. Its clinical feature ranges from asymptomatic to severe conditions such as myxedema coma [1]. The treatment of hypothyroidism with levothyroxine (LT4) is conventional in clinical practice and average daily doses of 100-200 *μ*g or 1.5-2.2 *μ*g/kg daily are utilized for replacement with TSH normalization or free T4 (FT4) elevation [2]. However, some patients develop refractory hypothyroidism due to malabsorption, pancreatic and liver disorders, drug interactions, polymorphisms in type 2 deiodinase, a high fiber diet, and poor compliance to daily oral LT4 replacement, which is reportedly the most common reason [3]. Although there have been no plausible recommendations for the treatment of refractory hypothyroidism, the weekly oral or intramuscular administration of LT4 may be useful [4, 5].

The intravenous administration of LT4 is commonly used for the medical emergencies such as myxedema coma [6]. However, to date, the safety and efficacy of the weekly intravenous and oral administration of LT4 as alternative ways to treat refractory hypothyroidism remain unknown. We herein precisely present the clinical course of the present case including pharmacokinetic data during the weekly intravenous and oral administration of LT4 and discuss the safety and efficacy of these treatments.

## 2. Case Presentation

A 41-year-old woman with refractory hypothyroidism was referred to our hospital. She was diagnosed with Graves' disease with thyrotoxicosis at the age of 36 years (thyroid weight: 51.1 g, TSH: <0.001 *μ*U/mL (reference range; 0.4 to 4.0), FT3: 21.85 pg/mL (reference range; 2.36 to 5.00). FT4: 4.00 ng/dL (reference range; 0.88 to 1.67), TSH receptor antibody (TRAb) 22.7 IU/L (reference range; < 2.0)). Neither methimazole, iodine potassium, nor three times of ^131^I ablation therapy ameliorated thyrotoxicosis. At the age of 39 years, she underwent total thyroidectomy, and oral LT4 replacement was initiated after surgery. Although the dose of LT4 was increased to 650 *μ*g/day (11.8 *μ*g/kg/day), with 150 *μ*g/day of liothyronine (LT3) also being administered, severe hypothyroidism persisted.

She had general fatigue, mild diarrhea, anasarca, hair loss, peripheral coldness, and dry skin. She was hospitalized for further examination to exclude malabsorption.

She was 161.6 cm in height and 55 kg in weight and had a body temperature of 36.3°C. Physical examinations showed a blood pressure of 101/67 mmHg with a heart rate of 56 beats/min, and laboratory findings revealed anemia, mild renal dysfunction, and hypercholesterolemia. Her serum TSH levels were extremely high as 146.8 *μ*IU/mL and FT3 and FT4 levels were undetectable (Table 1). Glucose tolerance (fasting plasma glucose of 71 mg/dL and HbA1c of 5.4%) and adrenal and pituitary hormone levels were within normal ranges. Autoimmune antibodies other than thyroid peroxidase antibody (TPOAb) were all negative (Table 1). Ultrasonic cardiography showed pericardial effusion with normal cardiac output, and electrocardiography revealed a very low voltage and mild bradycardia (data not shown). She was not receiving any concomitant medication that may have interfered with the absorption or metabolism of LT4 [7]. Her serum albumin levels were within normal range, suggesting no evidence of malabsorption (Table 1). Moreover, anemia and hypocalcemia prior to admission to our hospital were successfully treated by the administration of iron and calcium, respectively.* Helicobacter pylori *and other gastrointestinal tract infections were negative. A stool examination was normal. Upper and lower gastrointestinal endoscopies were performed and were also normal. Duodenal biopsy was performed and did not show pathological abnormalities consistent with malabsorptive disorder. After her admission to our hospital, the patient was handed LT4 tablets by nurses every morning; however, her intake was not confirmed.

**Figure 1:**
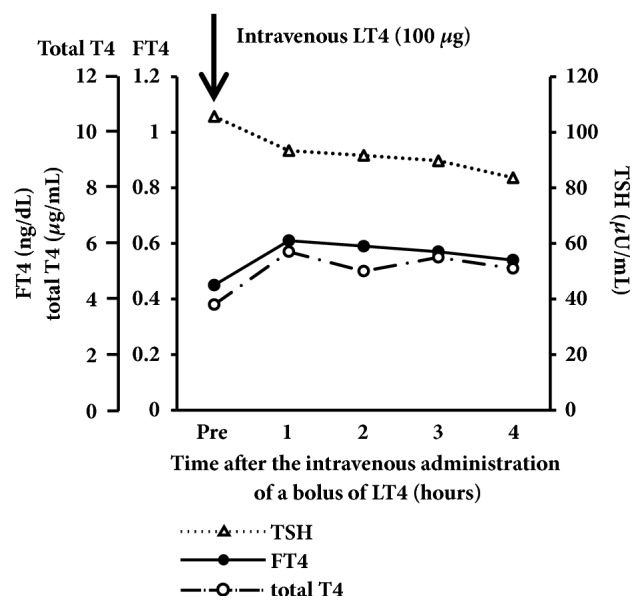
*Serum thyroid hormone and TSH levels after the intravenous administration of a single bolus of LT4 (100 μg)*. Serum FT4 and total T4 levels both increased, peaked 1 hour after the administration, and gradually decreased thereafter.

**Figure 2:**
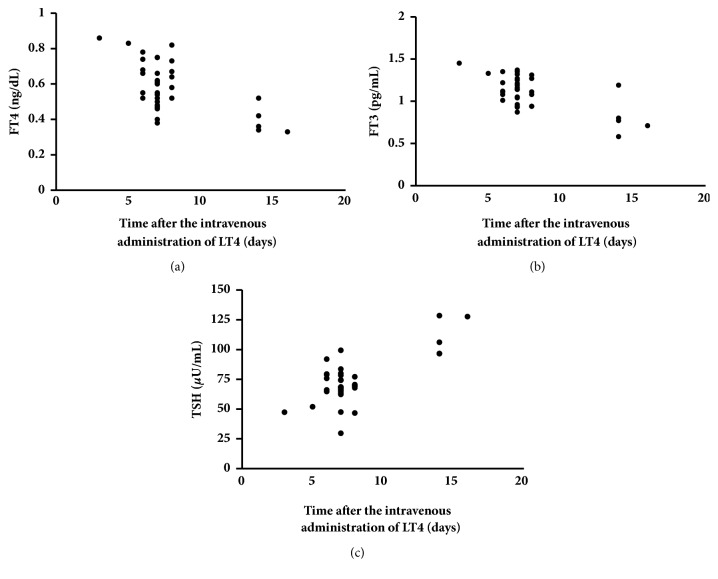
*Serum thyroid hormone and TSH levels after the intravenous administration of a single bolus of LT4 (300 μg)*. Serum FT4 (a), FT3 (b), and TSH (c) levels were measured several times of the administration.

**Figure 3:**
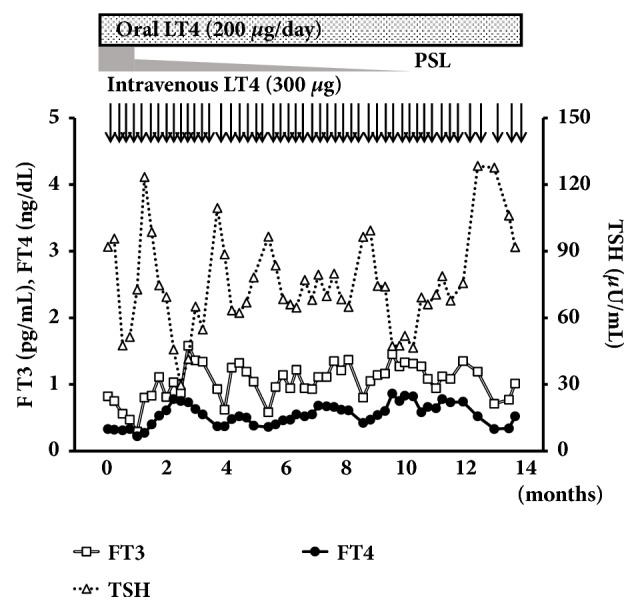
*LT4 (300 μg) intravenous administration once in a week and two weeks*. A daily oral LT4 (200 *μ*g/day) replacement was continued. PSL (20 mg/day) was also orally administered at the onset of the weekly intravenous administration of LT4 (300 *μ*g). It was tapered to 10 mg/day in a month after the administration. Thereafter, it was tapered by 1 mg/day per a month and subsequently discontinued 10 months after the intravenous administration of LT4. Twelve months after the intravenous administration of LT4, LT4 was intravenously administered once every two weeks, which resulted in the relapse of severe hypothyroidism with elevated serum TSH levels.

Since the additional rectal administration of LT4 suppository preparation (100 *μ*g/day) did not ameliorate hypothyroidism, we attempted its intravenous administration to treat refractory hypothyroidism under the permission and approval of the Ethical Committee of Tokyo Medical and Dental University Hospital and written informed consent was obtained from the patient and her father. An intravenous LT4 formula was dispensed in the Department of Pharmacy of the Tokyo Medical and Dental University hospital, following a previously reported prescription [8]. In brief, LT4 sodium salt pentahydrate (Sigma-Aldrich T2501, #6106-07-6) was dissolved by 0.1 N NaOH solution and diluted to a concentration of 200 *μ*g/2 mL by saline. A total of 300 *μ*g of LT4 was then diluted in 50 mL of saline and administered to the patient by intravenous drip infusion in 15 min. Since the patient had suffered from severe hypothyroidism for a long time, we did not really know whether her adrenal function was potentially normal or not. Therefore, prior to the intravenous administration of LT4, we administered 100 mg/day of hydrocortisone (HDC) in an intravenous drip to avoid relative adrenal insufficiency caused by rapid increases in thyroid hormone levels. Six days after the daily intravenous administration HDC, her thyroid hormone levels markedly improved. Therefore, we tapered oral administration of LT4 to 200 *μ*g/day and intravenously a bolus of LT4 was administered (100 *μ*g/day). Following the intravenous administration of a single bolus of LT4 (100 *μ*g/day), her serum FT4 levels were rapidly and markedly increased in 1 hour (Figure 1). At the time of discharge, we decided to administer 200 *μ*g/day of LT4 orally without the intravenous administration. Since we had already administered 100 mg/day of HDC in an intravenous drip for 10 days, we tapered oral administration of HDC to 20 mg/day upon the discharge. However, 7 days after her discharge, her thyroid hormone levels markedly decreased under the prescription. Thus, in the outpatient clinic, we administered 300 *μ*g of LT4 by intravenous bolus injection weekly for several weeks. Then, we examined the time course of serum FT4, FT3 and TSH levels for optimization (Figure 2). Serum FT4 and FT3 levels increased within 3 days of the administration and deceased thereafter (Figures 2(a) and 2(b)). Within seven days following the bolus intravenous administration, serum FT4 and FT3 levels remained mostly within normal range. Serum TSH levels increased again 14 days after the intravenous administration of a bolus of LT4 (300 *μ*g) in accordance with the decreases observed in serum FT4 and FT3 levels (Figure 2(c)). Based on these results, we selected the weekly intravenous administration of LT4 (300 *μ*g). Since then, her serum FT4 and FT3 levels had been maintained as low-normal with the weekly intravenous administration of LT4 for 14 months, whereas serum TSH levels had vary (Figure 3). At the time of the intravenous bolus administration of high dose LT4 (300 *μ*g) in the outpatient clinic, since HDC administration was already started, we decided to administer 20 mg/day of PSL orally instead of increasing the dose of HDC to avoid relative adrenal insufficiency. However, no symptom of adrenal insufficiency was found and her plasma ACTH and serum potassium levels decreased, possibly due to the administration of PSL. Therefore, we tapered the dose of PSL to 10 mg/day in a month after the onset of the weekly intravenous administration of LT4 (300 *μ*g). Thereafter, we carefully tapered the daily doses of PSL by 1 mg per month to avoid steroid withdrawal syndrome and subsequently withdrew PSL administration (Figure 3).

**Figure 4:**
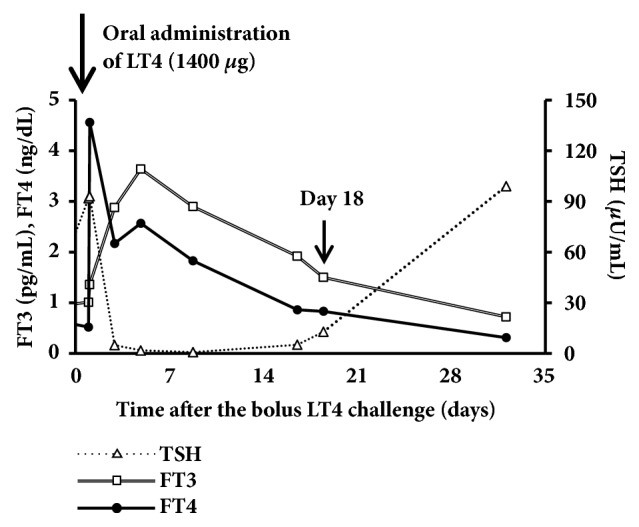
*Serum thyroid hormone and TSH levels after the oral administration of a single bolus of LT4 (1400 μg)*. Eighteen days after the administration, serum FT4 and FT3 levels were 0.83 ng/dL and 1.5 pg/mL, respectively. Her serum TSH levels were 12.78 *μ*IU/mL.

**Figure 5:**
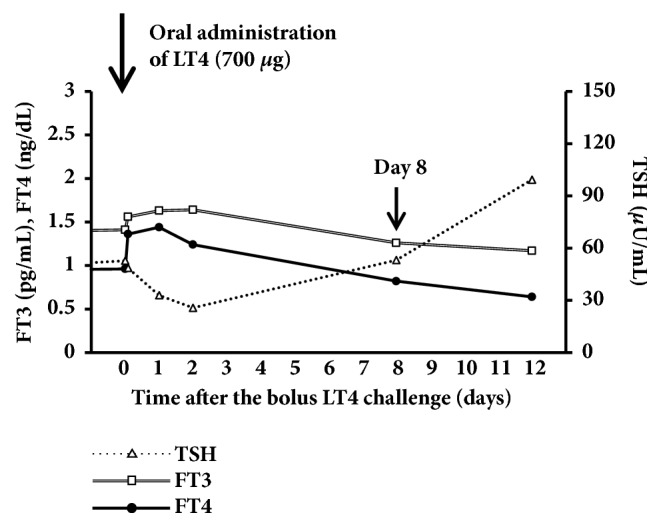
*Serum thyroid hormone and TSH levels after the oral administration of a single bolus of LT4 (700 μg)*. Eight days after the administration, serum FT4 and FT3 levels were 0.82 ng/dL and 1.26 pg/mL, respectively. Her serum TSH levels were 53.1 *μ*IU/mL.

Twelve months after weekly intravenous administration of LT4 (300 *μ*g), her hemoglobin levels increased, while serum creatinine, low-dense lipoprotein cholesterol, creatine kinase, and prolactin levels decreased to the normal range. No liver dysfunction or cardiovascular events were detected (Table 1). Under oral administration of 200 *μ*g/day of LT4, we attempted the intravenous administration of LT4 (300 *μ*g) once in two weeks, which resulted in the relapse of severe hypothyroidism, suggesting the pseudomalabsorption of LT4 due to poor compliance (Figure 3). We then attempted the single oral bolus administration (1400 *μ*g) under direct observation instead of the weekly intravenous administration of LT4 [3, 4]. The dose of LT4 selected was 7-fold the usual daily dose, which was 200 *μ*g/day [3, 4]. Following the oral administration of a single bolus of LT4, her serum FT4 levels rapidly elevated within 2 hours (0.52 to 4.56 ng/dL) as shown in Figure 4. Her serum FT4, FT3, and TSH levels were maintained within normal range for 15 days after the single bolus oral administration, and hypothyroidism relapsed thereafter (Figure 4). Based on the results, we employed 700 *μ*g of LT4 for a weekly oral administration protocol, which was 100 *μ*g of LT4 daily, because when we administered 1400 *μ*g of LT4, her FT4 levels after 2 hours were extremely high, which were clearly harmful and had remained elevated for more than one week. Under the weekly oral administration of 700 *μ*g of LT4, her serum FT4 and FT3 levels were elevated for 2 hours (FT4: 0.96 to 1.36 ng/dL, FT3: 1.41 to 1.56 pg/mL), and TSH levels decreased (52.8 to 48.6 *μ*IU/mL) without liver dysfunction or electrocardiogram abnormalities (Figure 5). Her serum FT4 and FT3 levels were maintained within normal ranges for 8 days after the single bolus oral administration, which demonstrates the relevance of the weekly oral administration of 700 *μ*g of LT4 (Figure 5).

Six months after the weekly oral administration of LT4, her renal and liver dysfunction and lipid profile improved (Table 1). We concluded that she developed severe hypothyroidism due to poor compliance to the daily oral LT4 replacement, even though she denied the poor compliance. Shi is currently being followed up at the psychiatric clinic. To date, her euthyroid status is maintained under the weekly oral administration of LT4 (700 *μ*g) for two years.

## 3. Discussion

We encountered a severe case of refractory hypothyroidism due to poor compliance to a daily oral LT4 replacement. In the present case, we initially suspected the malabsorption of thyroid hormone. Even though previous doctors gradually increased the dose of LT4, her thyroid hormone levels were not improved and her symptom caused by hypothyroidism was severe. Therefore, presumably followed by the treatment to myxedema coma [9], they decided to coadminister LT4 and LT3. An administration of high dose (150 *μ*g /day) of LT3, which might be potentially dangerous [9, 10], did not increase her free T3 levels (Table 1), suggesting that she was not taking medicine or that LT3 was not absorbed. However, the oral administration of LT4 (1400 *μ*g) under direct observation resulted in increase of serum FT4 levels, which excluded the malabsorption of LT4 and revealed poor compliance to the daily oral LT4 replacement [3].

When HDC was administered prior to the intravenous administration of LT4, her serum FT4 levels markedly increased, possibly because corticosteroid inhibits T4 to T3 conversion. However, the administration of PSL during the weekly intravenous administration of LT4 did not increase her serum FT4 levels.

Due to the relatively long half-life of LT4, the use of a weekly oral LT4 replacement is plausible [11], with previous studies reporting that the weekly oral administration of LT4 is a safe, well-tolerated, and effective therapy for patients with noncompliance [4, 5]. The treatment of hypothyroidism with weekly doses of LT4 has been shown to increase serum FT4 levels within approximately two hours and decrease FT4 levels immediately before the weekly dose [12], which is compatible with our results on the administration of a single bolus of LT4 (1400 *μ*g) (Figure 4). The present results suggest the efficacy, as well as safety of the oral administration of a single bolus of LT4 as a test for possible compliance issues. Our case also showed continuous stable thyroid function with 12.56 *μ*g/kg/week of LT4 being orally administered with no obvious adverse effects, such as liver dysfunction, angina, or thyrotoxicosis. The weekly oral administration of LT4 was previously reported to be safe [3–5, 12]. However, no large-scale clinical trials, particularly those including elderly patients and those with a history of ischemic heart disease, on weekly administration have been performed to date, and, thus, further evidence is required to prove the safety and efficacy of the weekly oral administration of LT4.

On the other hand, there have been no detailed clinical data on the weekly intravenous administration of LT4. Since the intravenous administration of LT4 once in two weeks resulted in severe hypothyroidism in the present case, the pharmacokinetics of LT4 via its intravenous administration may be similar to those via its oral administration [12]. In the present case, we demonstrated that the weekly intravenous administration of LT4 achieves stable normal thyroid hormone levels in the long term (14 months) without severe adverse effects. However, we only reported the results of one case that was treated with the weekly intravenous administration of LT4, which is a limitation. In the present study, we did not perform 24-h electrocardiogram monitoring, ultrasonic cardiography, or bone and muscle parameter measurements, which need to be assessed in future studies. Thus, concrete safety data on the weekly intravenous administration of LT4 were not obtained in the present study.

We treated a case of refractory hypothyroidism due to poor compliance with the weekly administration of LT4 both orally and intravenously, both of which were performed safely and effectively. We provided clinical data to suggest that the weekly intravenous administration of LT4 has potential as an alternative treatment to daily LT4 replacement for cases of refractory hypothyroidism with noncompliance. The oral administration of LT4 to patients on a daily basis represents an ideal treatment. However, the weekly oral or intravenous administration of LT4 may be an alternative for some patients with poor adherence such as the present case.

## Figures and Tables

**Table 1:** Changes in parameters following the intravenous (ivLT4) or oral administration of LT4.

Parameter	before ivLT4	after ivLT4	before oral LT4	after oral LT4
Systolic BP (mmHg)	101	142	125	104
Diastolic BP (mmHg)	67	94	78	67
Heart rate (bpm)	56	76	60	83
WBC (/*μ*L)	3400	4600	5000	5300
Hb (g/dL)	8.0	12.6	12.8	11.1
Ht (%)	25.3	37.7	39.4	35.3
PLT (x10000/*μ*L)	12.8	15.1	17.3	18.5
TP (g/dL)	6.6	6.2	7.2	6.9
Alb (g/dL)	3.7	4.3	3.8	3.3
BUN (mg/dL)	25	15	21	18
CRE (mg/dL)	1.29	0.75	1.07	0.97
UA (mg/dL)	5.0	4.3	5.2	4.9
Na (mEq/L)	141	139	141	139
K (mEq/L)	4.2	3.5	3.5	4.2
Cl (mEq/L)	108	106	108	104
Adjusted Ca (mg/dL)	8.3	9.0	8.9	9.1
IP (mg/dL)	4.4	3.9	3.1	3.3
LDH (U/L)	243	167	214	183
AST (U/L)	33	17	23	21
ALT (U/L)	22	13	18	16
*γ*-GTP (U/L)	28	11	14	12
ALP (U/L)	156	175	188	233
TG (mg/dL)	121	53	73	42
HDL-C (mg/dL)	49	78	109	97
LDL-C (mg/dL)	183	108	219	119
CK (IU/L)	206	55	108	64
CRP (mg/dL)	0.14	0.04	0.02	0.07
TSH (*μ*IU/mL)	146.8	69.22	147.4	34.23
FT3 (pg/mL)	<0.26	1.27	0.26	1.4
FT4 (ng/dL)	<0.02	0.58	0.07	0.68
Total T3 (ng/mL)	<10	42	29	63
Total T4 (*μ*g/mL)	<0.3	3.8	1.9	10.0
Tg (ng/dL)	<0.3	N/A	N/A	N/A
TBG (*μ*g/dL)	33.3	N/A	N/A	N/A
Tg Ab (IU/mL)	11 (ref.<28.0)	N/A	N/A	N/A
TPO Ab (IU/mL)	143 (ref.<16.0)	N/A	N/A	N/A
TR Ab (IU/L)	1.0 (ref.<2.0)	N/A	N/A	N/A
TS Ab (%)	149 (ref.<180)	N/A	N/A	N/A
Fe (*μ*g/dL)	58	65	43	31
UIBC (*μ*g/dL)	263	269	292	377
Ferritin (ng/dL)	38	38	8	7
Folic acid (mg/dL)	3.8	N/A	4.5	4.5
Vit.B12 (mg/dL)	N/A	N/A	1500	1500
GH (ng/mL)	1.53	2.67	N/A	1.68
LH (mIU/mL)	4.0	4.3	N/A	11.6
FSH (mIU/mL)	6.0	5.9	N/A	25.4
PRL (ng/mL)	37.8	8.7	N/A	13.0
ACTH (pg/mL)	25.6	13.2	N/A	17.8
Cortisol (*μ*g/dL)	13.3	10.0	N/A	6.8
IGF-I (ng/mL)	32	72	N/A	68

Parameters were evaluated before and 12 months after weekly intravenous LT4 (300 *μ*g) administration (ivLT4) or before and 6 months after weekly oral LT4 (700 *μ*g) administration (oral LT4). N/A: not applicable. ref: cutoff value or negative. Reference range: TSH; 0.4 to 4.0 *μ*U/mL, FT3; 2.36 to 5.00 pg/mL, FT4; 0.88 to 1.67 ng/dL.
